# Reduced urinary release of AQP1‐ and AQP2‐bearing extracellular vesicles in patients with advanced chronic kidney disease

**DOI:** 10.14814/phy2.15005

**Published:** 2021-08-26

**Authors:** Sayaka Oshikawa‐Hori, Naoko Yokota‐Ikeda, Hiroko Sonoda, Yosuke Sasaki, Masahiro Ikeda

**Affiliations:** ^1^ Department of Veterinary Pharmacology Faculty of Agriculture University of Miyazaki Miyazaki Japan; ^2^ Department of Nephrology Miyazaki Prefectural Miyazaki Hospital Miyazaki Japan; ^3^ Department of Animal and Grassland Sciences Faculty of Agriculture University of Miyazaki Miyazaki Japan

**Keywords:** aquaporin‐1, aquaporin‐2, chronic kidney disease, urinary extracellular vesicles

## Abstract

Although several studies have shown that release of water channel proteins, aquaporin 1 (AQP1) and AQP2 in urinary extracellular vesicles (uEV‐AQP1 and ‐AQP2), were altered in experimental kidney injury models, their release in human chronic kidney disease (CKD) has been largely unexplored. The aim of the present study was to clarify whether the release of uEV‐AQP1 and ‐AQP2 is altered in patients with CKD. Urine samples were collected from 15 healthy volunteers (normal group) and 62 CKD patients who were categorized into six glomerular filtration rate (GFR) categories (G1, G2, G3a, G3b, G4, and G5) in between 2005 and 2016 at Miyazaki Prefectural Miyazaki Hospital, Japan. uEV‐proteins were evaluated by immunoblot analysis. The release of AQP1 and AQP2 were significantly decreased in patients with both CKD G4 and G5, in comparison with the normal group. The area under the receiver operating characteristic (ROC) curve (AUC) values for AQP1 and AQP2 in patients with CKD G4 and G5 were 0.926 and 0.881, respectively. On the other hand, the AUC values in patients with CKD G1‐G3 were 0.512 for AQP1 and 0.680 for AQP2. Multiple logistic regression analysis showed that AQP1 and AQP2 in combination were useful for detecting CKD G4 and G5, with a higher AUC value of 0.945. These results suggest that the release of uEV‐AQP1 and ‐AQP2 was decreased in patients with CKD G4 and G5, and these proteins might be helpful to detect advanced CKD.

## 1 INTRODUCTION

Chronic kidney disease (CKD) is a significant public health concern worldwide (Sarnak et al., 2003), especially in view of its markedly high morbidity. For example, the overall prevalence of CKD in the general population of the United States is about 14% (https://www.niddk.nih.gov/health‐information/health‐statistics/kidney‐disease). Patients with CKD frequently show fluid overload (Hung et al., 2015).

Extracellular vesicles (EVs) that include exosomes (small EVs) and microvesicles have been identified in various type of biological fluids such as serum, urine, milk, and saliva, and a focus of intense translational research to identify novel biomarkers (Colombo et al., 2014; Pisitkun et al., 2004). Urinary EVs (uEVs) have attracted attention because they contain various types of renal functional proteins derived from specific and different regions of the nephron, including Na^+^/H^+^ exchanger isoform 3, aquaporin 1 (AQP1), AQP2, sodium‐potassium‐chloride co‐transporter 2, and sodium‐chloride cotransporter, suggesting that these renal proteins in uEVs could provide information on renal disease states (Gonzales et al., 2009; Oshikawa et al., 2016; Pisitkun et al., 2006). However, the usefulness of proteins in uEVs for monitoring patients with CKD has been largely unexplored.

So far, we have shown that the release of AQP1‐ and AQP2‐bearing uEVs (uEV‐AQP1 and ‐AQP2) are altered in experimental kidney injury models such as gentamicin (Abdeen et al., 2014), cisplatin (Sonoda et al., 2019), puromycin aminonucleoside (Abdeen et al., 2020), and ischemia/reperfusion (I/R) models (Asvapromtada et al., 2018). However, the release of uEV‐AQP1 and ‐AQP2 has not yet been investigated in human kidney diseases.

In the present study we investigated whether release of uEV‐AQP1 and ‐AQP2 was altered and determined the state of CKD. We also assessed other proteins in uEVs, including tumor susceptibility gene 101 (TSG101) protein, apoptosis‐linked gene 2‐interacting protein X (Alix), and Tamm‐Horsfall protein (THP), all of which are reported to be possible uEV marker proteins (Fernández‐Llama et al., 2010; Soo et al., 2012; Street et al., 2011; Yang et al., 2012).

## 2 METHODS

### 2.1 Study participants and design

All samples were obtained using study protocols approved by the Miyazaki Prefectural Miyazaki Hospital Institutional Review Board and University of Miyazaki Department Institutional Review Board in accordance with the Ethical Guidelines for Clinical Studies in Japan.

CKD patients were diagnosed by biopsy or ultrasonography at Miyazaki Prefectural Miyazaki Hospital Institution from 2005 through 2016. A eGFR was calculated using the Japanese GFR equation based on serum creatinine: for males, eGFR = 194 × Cr −1.094 ml/min/ 1.73 m^2^ × age −0.287, and for females, eGFR = 194 × Cr −1.094 × age −0.287 × 0.739. In accordance with the KDIGO guidelines, patients were grouped into six CKD GFR categories. The history of medication in patients is shown in Table S1.

We finally recruited healthy male subjects who yielded normal results of the urine dipstick test, and 15, 12, and 10 individuals were used as the normal groups for the examination of AQP1 and AQP2, TSG101, and Alix and THP, respectively. The reason for this difference in numbers was the paucity of the sample.

### 2.2 Analysis of blood and urine parameters

Urinary creatinine concentration was measured using an autoanalyzer (Fuji Film Medical). Urinary osmolality was measured using an automatic osmometer (Osmostation om‐6060, Arkray).

### 2.3 Collection of urine samples and isolation of urinary extracellular vesicles

First midstream urine of the day was collected in the morning from each CKD patient. The procedure for isolation of uEVs was performed as described previously (Oshikawa‐Hori et al., 2019). Briefly, just after the urine collection, a protease inhibitor mixture (1 mM EDTA, 1 mM p‐amidinophenyl methanesulfonyl fluoride hydrochloride, and 10 µg/ml leupeptin for final concentrations) was added to the collection tube. The collected urine was centrifuged at 1,000 *g* at 4°C for 15 min and the supernatant was centrifuged at 17,000 *g* at 4°C for 30 min. Thereafter, the supernatant was mixed with a protease inhibitor mixture (Complete protease inhibitor cocktail tablet, Roche Diagnostics, Rotkreuz, Switzerland) followed by ultracentrifugation at 200,000 *g* (Optima TL Ultracentrifuge; Beckman Instruments, CA) at RT for 1 h using a thickwall polycarbonate tube (#355630, Beckman Instruments) and the MLA‐55 rotor (k factor = 54, Beckman Instruments). The resulting pellet (a fraction rich in EVs) was suspended in a solution containing a protease inhibitor mixture. The suspension was then mixed with 4× sample buffer (8% SDS, 50% glycerol, 250 mM TrisCl, 0.05% bromophenol blue, 200 mM DTT, pH 6.8) and subsequently, the sample was incubated at 37°C for 30 min. The final samples were stored at −80°C until the use.

In EV characterization experiments (Thery et al., 2018), nanoparticle tracking analysis showed that the size distribution of vesicles in our EV fraction had an averaged mode of around 78 nm and a standard deviation of around 55 nm. Also, as judged by immunoblot analysis, our fraction contained CD9, TSG‐101, and Alix, indicating that the fraction was rich in EVs.

### 2.4 Gel electrophoresis and immunoblot analysis

The loading amounts of protein samples were adjusted to equalize the total amount of urinary creatinine for each lane. It is very difficult to determine the normalization method in uEV studies (Thery et al., 2018). Since creatinine is normally excreted in urine at a steady rate, the normalization method has been considered (Thery et al., 2018). Also, in a preliminary experiment, we observed that there was a good correlation between the amount of creatinine and the level of uEV‐AQP1 or ‐AQP2 (Figure S1). Therefore, in this study we employed the normalization method.

The antibodies used were as follows: anti‐AQP1 antibody (catalog no. sc‐20810, Santa Cruz Biotechnology), anti‐AQP2 antibody (catalog no. sc‐9882, Santa Cruz Biotechnology), anti‐TSG101 antibody (catalog no. ab83‐100, Abcam, Cambridge, UK), anti‐Alix antibody (catalog no. sc‐49268, Santa Cruz Biotechnology), and anti‐THP antibody (catalog no. sc‐20631, Santa Cruz Biotechnology), anti‐rabbit IgG (catalog no. cs‐7074, Cell Signaling Technology), anti‐mouse IgG (catalog no. 1858413, Thermo Fisher Scientific), and anti‐goat IgG (catalog no. P0449, Dako Japan). The antibody‐antigen reaction was visualized by using a Super Signal chemiluminescence detection system (Thermo Fisher Scientific) and the detected signal was quantified using the software package Win Roof software V5.7 (Mitani Corporation, Tokyo, Japan) or ImageQuant TL software (GE Healthcare).

We always loaded one constant control sample comprising a mixture of the samples from three healthy individuals in the same gel, and the resulting band intensity was expressed as a percentage of the constant control band intensity.

### 2.5 Statistical analysis

Box plots were created by using the BoxPlotR (http://boxplot.tyerslab.com) (Spitzer et al., 2014) and statistical comparisons between the groups were performed by Steel‐Dwass test using the Mephas (http://www.gen‐info.osaka‐u.ac.jp). Statistical significance was accepted for all tests at *p* < 0.05. Receiver operating characteristic (ROC) curves were generated and the sensitivity and the specificity of the cut‐off values were calculated using the StatFlex software package (version 6.0, Artech). The results were also confirmed using Easy R (Kanda, 2013). Multiple logistic regression analysis was also performed using StatFlex software.

## 3 RESULTS

### 3.1 Characteristics of study participants

The clinical and laboratory parameters of patients examined in this study are shown in Table 1.

**TABLE 1:** Clinical characteristics of the patients

GFR category	G1	G2	G3a	G3b	G4	G5
Patients (n)	8	13	13	9	6	13
Sex (men/women)	2 / 6	6 / 7	4 / 9	5 / 4	3 / 3	5 / 8
Age, median	22.5	39.0	63.0	51.0	61.0	57.0
Age, range	18–65	17–72	42–76	40–80	52–76	25–72
BUN, median	8.7	9.8	13.6	18.8	32.2	77.1
BUN, range	7.1–14.8	6.8–15.2	8.7–24.7	12.8–24.3	18.8–46.2	41.9–83.5
SCr, median	0.6	0.7	0.9	1.3	2.6	8.1
SCr, range	0.5–0.8	0.6–1.0	0.8–1.3	1.1–1.9	1.4–2.9	4.1–10.3
Urinary osmolality (n), mean	493 (6)	579 (10)	459 (12)	346 (8)	324 (6)	280 (10)
Urinary osmolality, range	343–929	230–1097	152–1096	262–601	138–533	128–333

Values other than patient numbers represent the median and the range. The number of patients for whom osmolality was measured, as shown in parenthesis (9th row), was smaller than that of the patients overall (1st row) due to the paucity of the sample. The causes of CKD are as follows: G1, IgA nephropathy (IgA) (n = 5), focal segmental glomerulosclerosis (FSGS) (1), membranous nephropathy (MN) (2); G2, IgA (6), non‐IgA mesangial proliferative glomerulonephritis (non‐IgA MPGN) (1), diabetic nephropathy (DN) (2), FSGS (1), purpura nephritis (PN) (2), MN (1); G3a, IgA (2), non‐IgA MPGN (1), obesity‐related glomerulopathy (ORG) (1), minimal change disease (MCD) (3), FSGS (1), PN (1), MN (3), autosomal dominant polycystic kidney disease (ADPKD) (1); G3b, ANCA‐related glomerulonephritis (ANCA) (2), IgA (4), ORG (1), MCD (1), FSGS (1); G4, ANCA (1), IgA (4), ADPKD (1); G5, end stage renal disease (10), ANCA (2), crescentic glomerulonephritis (1).

### 3.2 Protein analysis in uEVs

**FIGURE 1:**
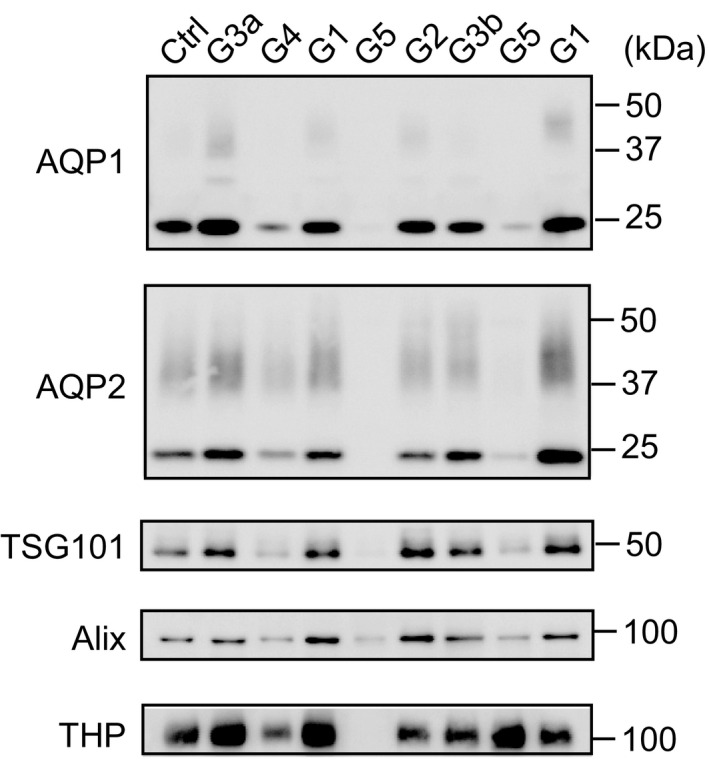
Representative immunoblot results of AQP1, AQP2, TSG101, Alix, and THP in urinary extracellular vesicles (uEVs). From the left, samples from a control (Ctrl) and from patients with G3a, G4, G1, G5, G2, G3b, G5, and G1 were loaded. Each sample was loaded with the same amount of creatinine (250 µg/lane for AQPs, 500 µg/lane for TSG101 and Alix, and 125 µg/lane for THP)

**FIGURE 2:**
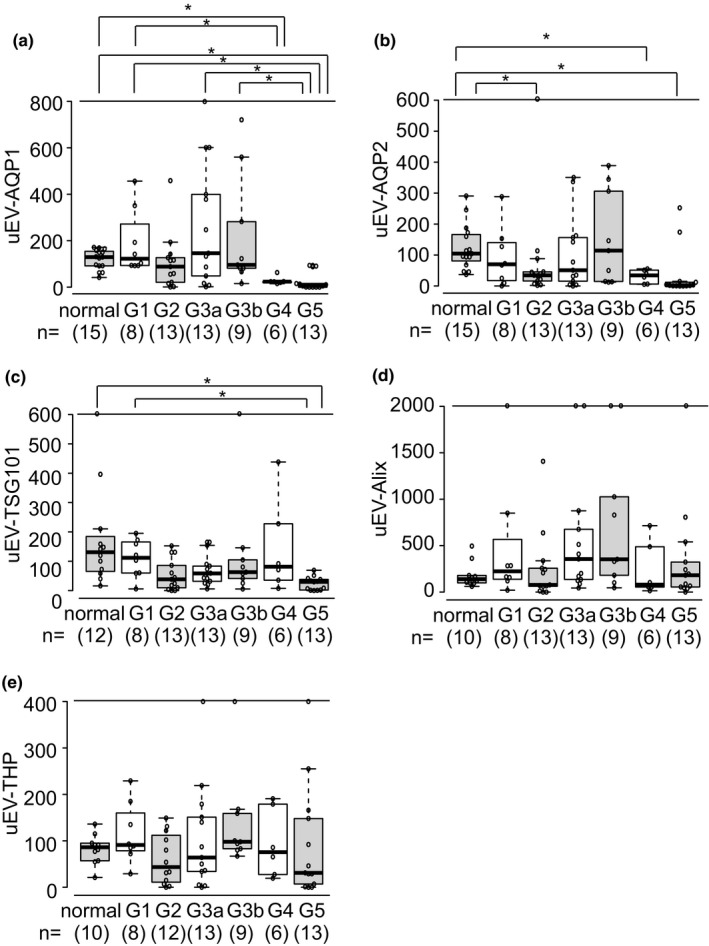
uEV‐protein levels in CKD patients. Dot and boxplots of uEV‐AQP1 (a), ‐AQP2 (b), ‐TSG101 (c), ‐Alix (d), and ‐THP (e) are shown. The thick line of the box plots indicates the median, and the top and bottom borders show the 25th and 75th percentiles. The whiskers represent 1.5 times the IQR from the lower and upper quartiles (Tukey). Data points beyond the Y axis maximum plot are shown on the upper line of the graph. Quantitative data were obtained from immunoblot analysis. *p <0.05 compared among each CKD category and the normal group (Steel‐Dwass test)

Figure 1 shows typical immunoblot images of uEV‐AQP1, ‐AQP2, ‐TSG101, ‐Alix, and ‐THP in patients and the control, and Figure 2 shows the summarized data. Also, all original blots were shown in Figure S2–S6.

In patients with CKD G5, the levels of the uEV‐AQP1 (4.9%, interquartile range (IQR), 0%, 15.6%), ‐AQP2 (2.1%, IQR, 0.5%, 12.1%), and ‐TSG101 (29.7%, IQR, 1.9%, 38.1%) were significantly lower than those in the normal group (AQP1, 129.3%, IQR, 91.6%, 154.2%; AQP2, 104.7%, IQR, 81%, 166.3%; TSG101, 130.1%, IQR, 65.5%, 184.4%), respectively. In patients with CKD G4, the levels of uEV‐AQP1 and ‐AQP2 were also significantly decreased to 23.5% (IQR 20%, 24%) and to 34.2% (IQR 6.2%, 51.2%), respectively, but no significant change for uEV‐TSG101 (81.3%, IQR, 35.5%, 227.7%) was observed. The levels of uEV‐Alix and ‐THP did not differ significantly among the groups.

We also checked the relationship between urine osmolality and uEV‐AQP1 or ‐AQP2. No significant correlations were obtained (urine osmolality vs. uEV‐AQP1, r = −0.015, n = 52; urine osmolality vs. uEV‐AQP2, r = 0.106, n = 52).

**FIGURE 3:**
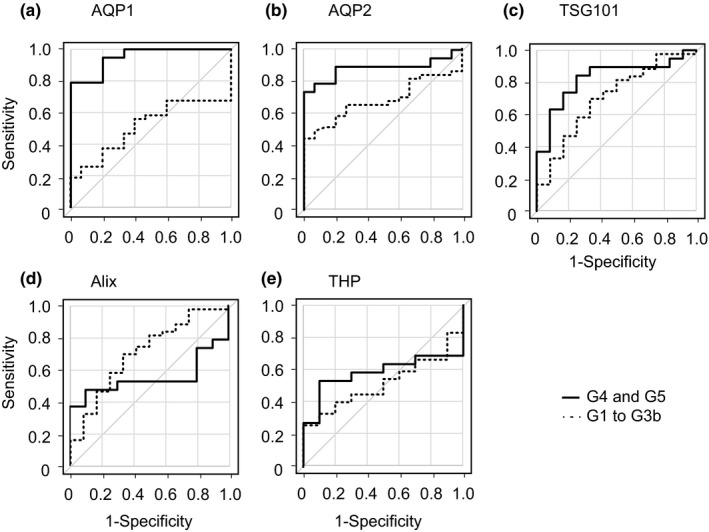
Receiver operating characteristic (ROC) curve analysis of uEV‐proteins in CKD patients. ROC curves of uEV‐AQP1 (a), ‐AQP2 (b), ‐TSG101 (c), ‐Alix (d), and ‐THP (e) are shown

Next, ROC curve analysis was performed to examine the diagnostic accuracy of these uEV‐proteins for the patients. Examination of uEV‐AQP1 or ‐AQP2 in the 19 patients with CKD G4 and G5, and those in the 15 normal healthy volunteers yielded a ROC AUC of 0.951 for AQP1 (95% confidence interval (CI), 0.888 to 1) and 0.884 for AQP2 (95% CI: 0.757–1) (Figure 3). AQP1 had 78.9% of sensitivity and 100% specificity with cutoff value of 24.0%, and AQP2 had 73.7% sensitivity and 100% specificity with cutoff value of 28.8%. On the other hand, for uEV‐TSG101, the AUC was 0.825 (95% CI: 0.67–0.979), the sensitivity 84.2%, and the specificity 75.0% with cutoff value of 70.9%. The AUC values for uEV‐Alix and ‐THP were 0.547 (95% CI: 0.332–0.762) and 0.579 (95% CI: 0.365–0.792), respectively.

We also performed ROC curve analysis for the patients with CKD G1 to G3. This yielded AUC values for AQP1, AQP2, TSG101, Alix, and THP of 0.512 (95% CI: 0.362–0.662), 0.680 (95% CI: 0.546–0.814), 0.703 (95% CI: 0.532–0.875), 0.623 (95% CI: 0.463–0.784), and 0.521 (95% CI: 0.359–0.683), respectively (Figure 3).

**FIGURE 4:**
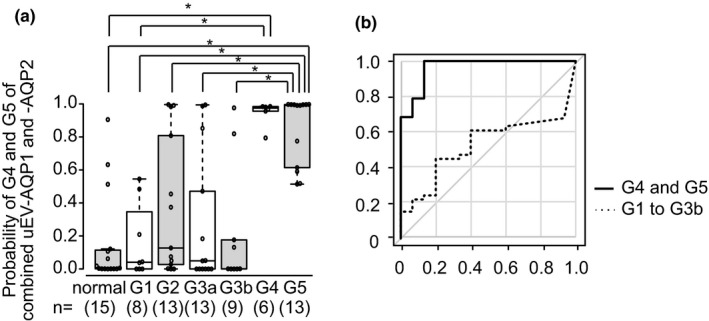
Multiple logistic regression analysis for uEV‐AQP1 and ‐AQP2. A dot and boxplot (a) and the corresponding ROC curve (b) for the predicted probabilities of G4 and G5 are shown. *p < 0.05 compared among each CKD category and normal (Steel‐Dwass test). The AUC of the ROC curve is 0.965 for G4 and G5 (95% CI, 0.911 −1) and 0.522 (95% CI: 0.37–0.675) for G1 to G3b

As the AUC values for uEV‐AQP1, ‐AQP2, and ‐TSG101 for patients with CKD G4 and G5 were high (>0.8) (El Khouli et al., 2009; Metz, 1978), in order to check whether the combination of these proteins would yield better accuracy than for each protein alone, we employed a multiple logistic regression model. Using a stepwise approach, only a combination of AQP1 and AQP2 was selected (default setting; *p*‐value cut‐off point of 0.15). The predicted probabilities (P) for uEV‐AQP1 and ‐AQP2 in combination were calculated by equations of 1 / (1 + e^−x^) and x = 5.66277 − 0.0591 * (AQP1) – 0.0210 * (AQP2). The summarized data are shown in Figure 4. The P value for either CKD G4 or G5 was significantly increased in comparison with that for the normal group. Furthermore, ROC curve analysis using that P value produced an AUC value of 0.965 (95% CI: 0.911–1), a sensitivity of 100%, and a specificity of 88.7% with a cutoff value of 0.514.

## 4 DISCUSSION

Our present study showed that release of uEV‐AQP1 and ‐AQP2 was significantly decreased in patients with CKD G4 and G5, in comparison with those of the control group. The alteration seen in the release of uEV‐TSG101 was similar to that of uEV‐AQP1 and ‐AQP2, but the decrease in patients with CKD G4 did not reach statistical significance. The release of uEV‐Alix and ‐THP did not differ among the patient groups. ROC analyses revealed that the AUC values for the release of uEV‐AQP1 and ‐AQP2 in patients with CKD G4 and G5 were 0.951 and 0.884, respectively, and the two in combination had a higher AUC value of 0.965. It has been reported that the AUC values of more than 0.9 and 0.8–0.9 are considered to represent excellent and good biomarkers, respectively (El Khouli et al., 2009), and therefore release of uEV‐AQP1 and ‐AQP2, and two in combination, could be used as biomarkers to detect advanced CKD such as CKD G4 and G5.

The mechanism underlying the decreases in the release of uEV‐AQP1 and ‐AQP2 in the patients with advanced CKD was currently unclear. One factor that has been reported to determine the release of uEV‐proteins is the level of renal protein expression (Oshikawa et al., 2016). In fact, in several experimental models it has been reported that the release of uEV‐AQP1 and ‐AQP2 was decreased along with that of their renal expression levels (Abdeen et al., 2014, 2020; Asvapromtada et al., 2018; Sonoda et al., 2019). Although no study on the relationship between human renal and uEV‐AQPs has been reported, the decrease in the renal expression of AQP1 in patients with pediatric congenital hydronephrosis has been shown to be dependent on the degree of renal dysfunction (Li et al., 2012). These findings suggest that the decreased release of uEV‐AQP1 and ‐AQP2 in patients with advanced CKD might be due to reduction of their renal expression.

EVs include exosomes and microvesicles, and in the present study we examined marker proteins of uEVs including TSG101, Alix, and THP. The endosomal sorting complex required for transport (ESCRT) machinery is thought to be important for the biogenesis of multivesicular endosomes, containing intracellular vesicles that become exosomes. The ESCRT machinery comprises four protein complexes: ESCRT‐0, ‐I, ‐II, and ‐III. TSG101 is a component of ESCRT‐I and Alix is an adaptor protein in the ESCRT machinery (Bissig & Gruenberg, 2014; Colombo et al., 2014). THP is a glycosylphosphatidylinositol‐linked membrane protein present mainly in the thick ascending limb of Henle's loop, and is the most abundant soluble protein in urine (Pisitkun et al., 2006). Here we found that the release patterns of uEV‐TSG101, ‐Alix, and ‐THP differed among the patients. Currently, the reason for this difference is unclear. So far, it has been reported that TSG101 might be EV (exosome)‐specific (Koritzinsky et al., 2019; Morrison et al., 2016) and that Alix, in addition to its presence on exosomes, might also be found on microvesicles, which are larger in size than exosomes (Colombo et al., 2014). THP is considered to be a major component of non‐EV co‐isolated structures (Street et al., 2011; Thery et al., 2018). Therefore, differences in EV‐specificity might be one possible reason for the variations of the release pattern.

Biomarkers capable of detecting CKD G4 and G5 would improve and support the evaluation of renal dysfunction at the time of clinical diagnosis. The management and therapeutic strategy for patients with CKD G4 and G5 are stricter than those for patients whose disease is less severe. For example, according to the guidelines for Kidney Disease Improving Global Outcomes (KDIGO), the recommended protein intake for patients with CKD G4 and G5 has been <0.8 g/kg/day (KDIGO Board members, 2013). Also, a protein intake of 0.55 g/kg/day in patients with CKD G4 and G5 reportedly allowed better metabolic control and reduced the need for medication (Cianciaruso et al., 2008). The KDIGO guidelines (KDIGO Board members, 2013) stipulate that patients with CKD G4 and G5 are required to have more frequent checks for anemia (at least twice a year) than those with less severe disease. In addition, the use of gadolinium contrast examination is also limited in these patients as well as administration of a number of agents including beta‐blockers, non‐steroidal anti‐inflammatory drugs, macrolides, sulfonylureas, insulin, metformin, lipid‐lowering statins, cisplatin, low‐molecular‐weight heparins, and warfarin. Given these facts, discrimination of CKD G4 and G5 from CKD G3 or less is profoundly important from the viewpoint of management and therapy.

This study had several limitations. A larger research population would have been desirable, especially as only six of the participants had CKD G4, making it more difficult to obtain the precise AUC value and significance relative to the normal group. We did not study the level of renal protein expression in CKD patients, which meant we were unable to investigate the mechanism underlying the decreased release of uEV‐AQP1 and ‐AQP2. Establishment of a more accurate measurement method would be essential for determining the cut‐off values for uEV‐AQP1 and ‐AQP2, since immunoblot analysis is a semi‐quantitative approach. Furthermore, in our study the relationship between release of uEV‐AQPs and proteinuria or treatment history of patients could not be examined, and these points should be examined by increasing the number of cases in the future.

In conclusion, this study has demonstrated that reduction of uEV‐AQP1 and ‐AQP2 was associated with advanced CKD. ROC analysis revealed that uEV‐AQP1 and ‐AQP2 reflected CKD progression of G4 and above, and particularly, the use of both uEV‐proteins in combination yielded better results than the use of either protein alone. Overall, these findings suggest that uEV‐AQP1 and ‐AQP2 may be applicable as novel biomarkers for diagnosis of advanced CKD.

## CONFLICT OF INTEREST

We have no financial interest to disclose.

## AUTHOR CONTRIBUTIONS

S.O.‐H., N.Y.‐I., and M.I. conceived and designed the research; S.O.‐H., N.Y.‐I., and M.I. performed experiments; S.O.‐H., H.S., Y.S., and M.I. analyzed the data; S.O.‐H., N.Y.‐I., Y.S., and M.I. interpreted the experimental results; S.O.‐H., H.S., and M.I. prepared figures; S.O.‐H. drafted manuscript; S.O.‐H. and M.I. edited and revised manuscript; S.O.‐H., N.Y.‐I., H.S., Y.S., and M.I. approved final version of manuscript.

## Supporting information



Fig S1

Fig S2‐S6

Table S1
