# Supplementary material for: Reduced urinary release of AQP1‐ and AQP2‐bearing extracellular vesicles in patients with advanced chronic kidney disease
Source: Physiol Rep. 2021 Aug 26;9(17):e15005. doi: 10.14814/phy2.15005 (PMC8387789; doi:10.14814/phy2.15005)
Supplement: Supplementary file 1 — Fig S1 [file PHY2-9-e15005-s002.pdf]

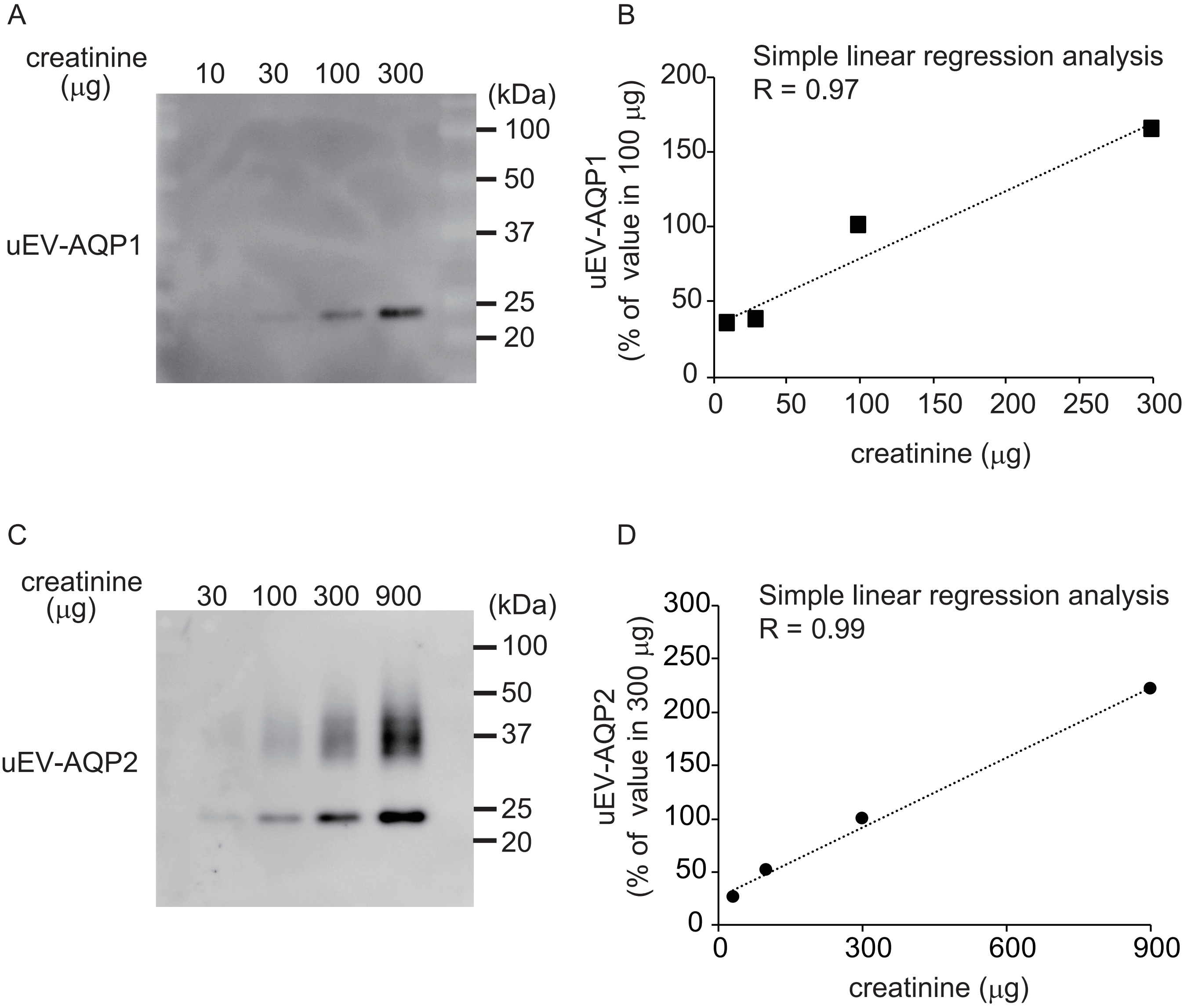

suppl Fig. 1. The relationship between the amounts of urinary creatinine and uEV-AQP1 (A & B) or -AQP2 (C & D).
A and C: Typical immunoblots are shown.
C and D: Immunoblotting results were quantified and the results of corelation analysis are shown.
Each value is expressed as a percentage of the result with 100 μg (B) or 300 μg (D) total creatinine. R in B or D represents the correlation coefficient.
The line is the least-squares regression line.
