# Supplementary material for: Reduced urinary release of AQP1‐ and AQP2‐bearing extracellular vesicles in patients with advanced chronic kidney disease
Source: Physiol Rep. 2021 Aug 26;9(17):e15005. doi: 10.14814/phy2.15005 (PMC8387789; doi:10.14814/phy2.15005)
Supplement: Supplementary file 2 — Fig S2‐S6 [file PHY2-9-e15005-s001.pdf]

suppl Fig. 2 AQP1

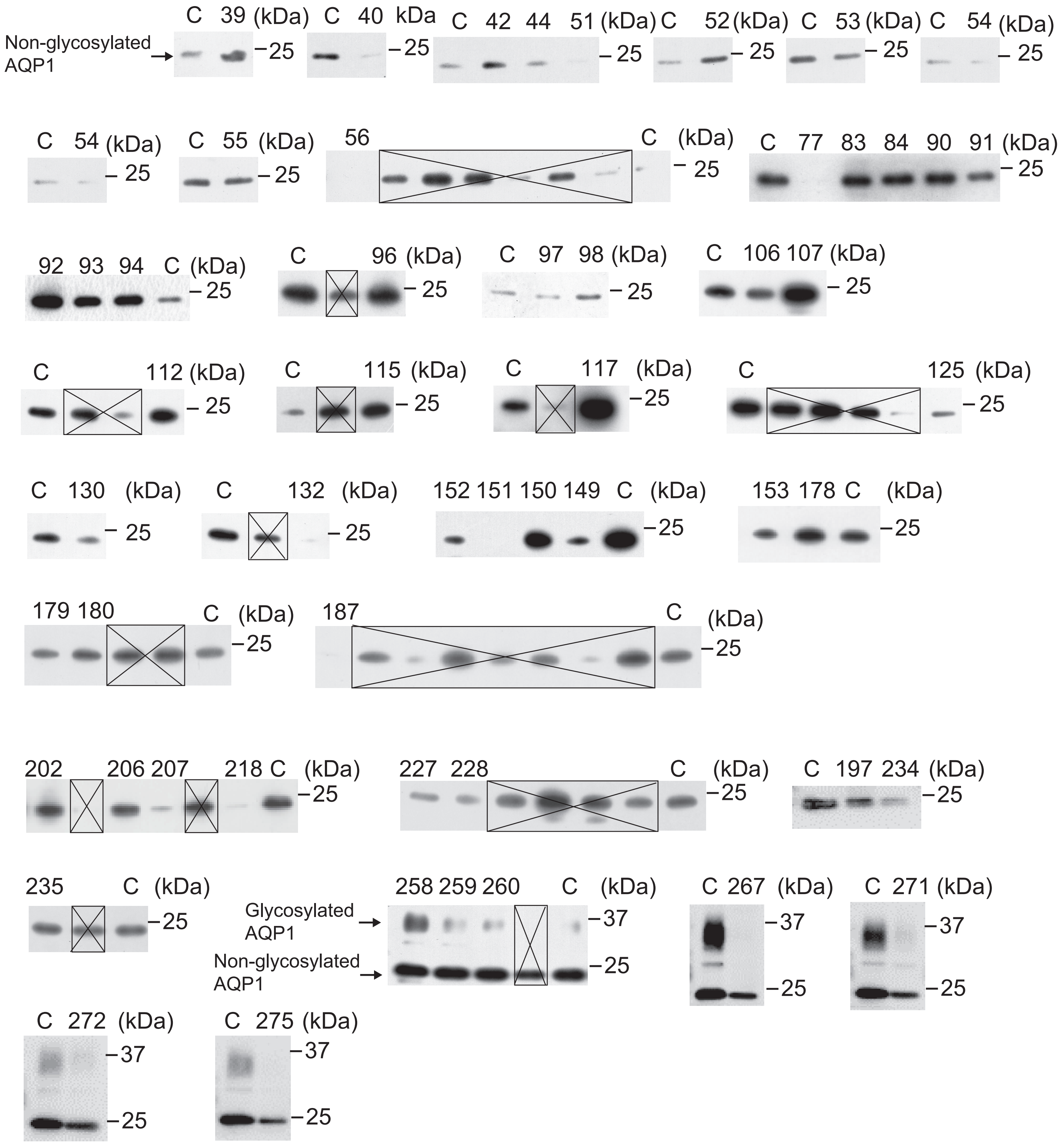

C: control

G1 39, 55, 84, 97, 98, 206, 208 (Figure1), 252 (Figure1)

G2 51, 53, 54, 91,107, 117, 150, 153, 178, 218, 229 (Figure1), 258, 260

G3a 52, 90, 93, 94, 106, 112, 115,130, 180, 201 (Figure1), 227, 228, 272

G3b 42, 83, 92, 125, 149, 179, 235, 243 (Figure1), 259

G4 197, 203 (Figure1), 207, 234, 271, 275

G5 40, 44, 56, 77, 96, 132, 151, 152, 187, 202, 220 (Figure1), 245 (Figure1), 267

The cross mark is a sample (are samples) from other than the subject(s) of this research.

suppl Fig. 3 AQP2

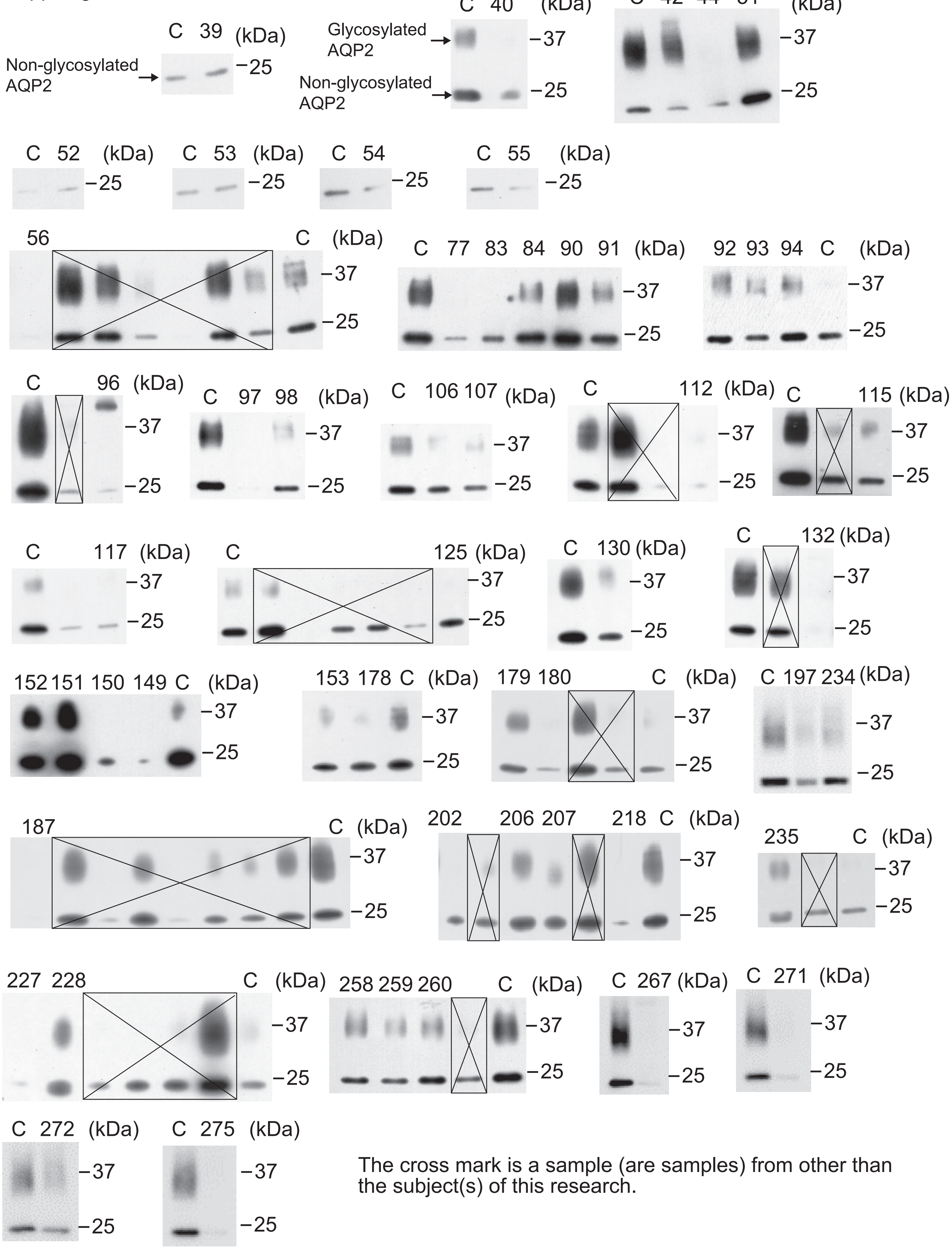

The cross mark is a sample (are samples) from other than the subject(s) of this research.

suppl Fig. 4 TSG101

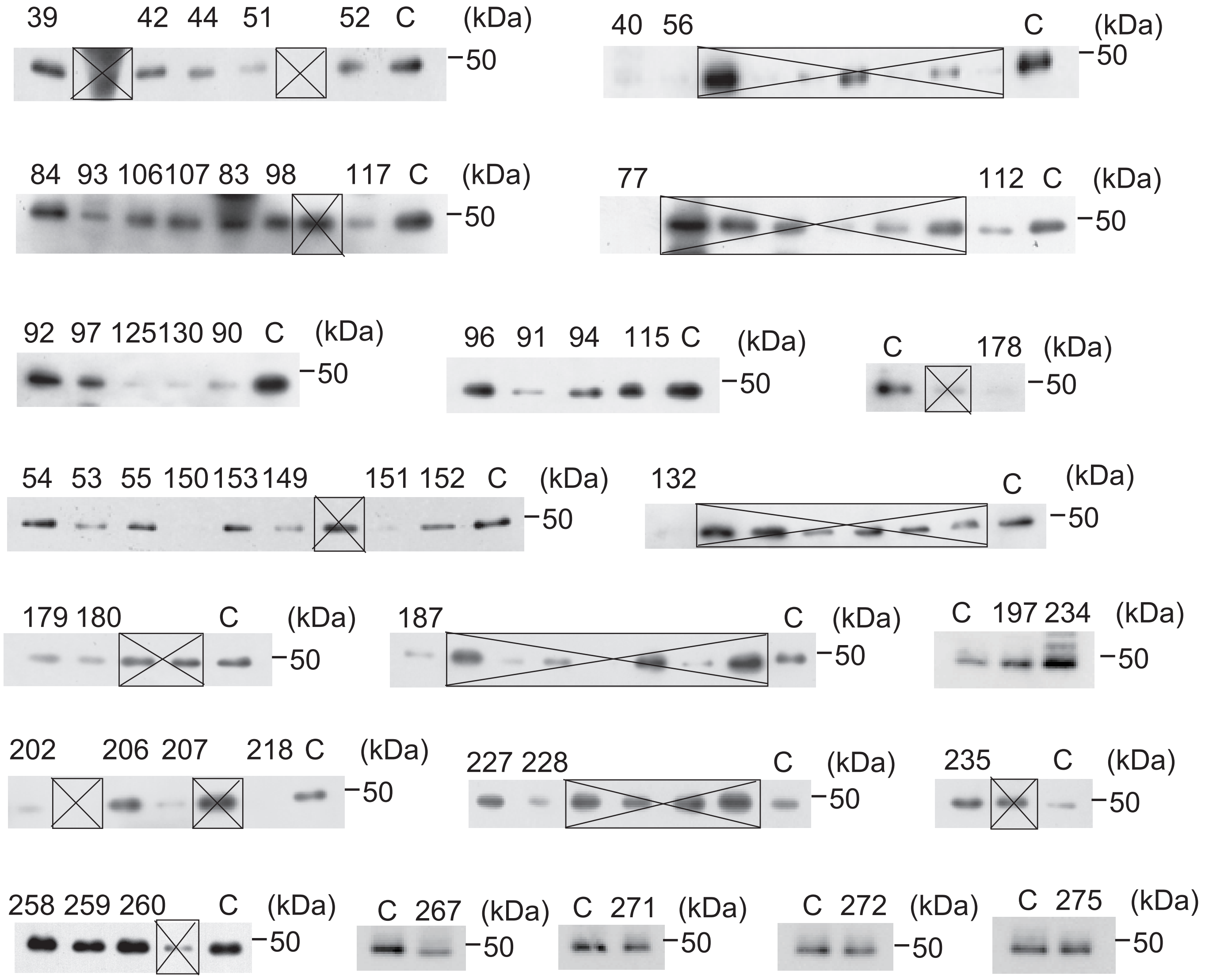

The cross mark is a sample (are samples) from other than the subject(s) of this research.

suppl Fig. 5 Alix

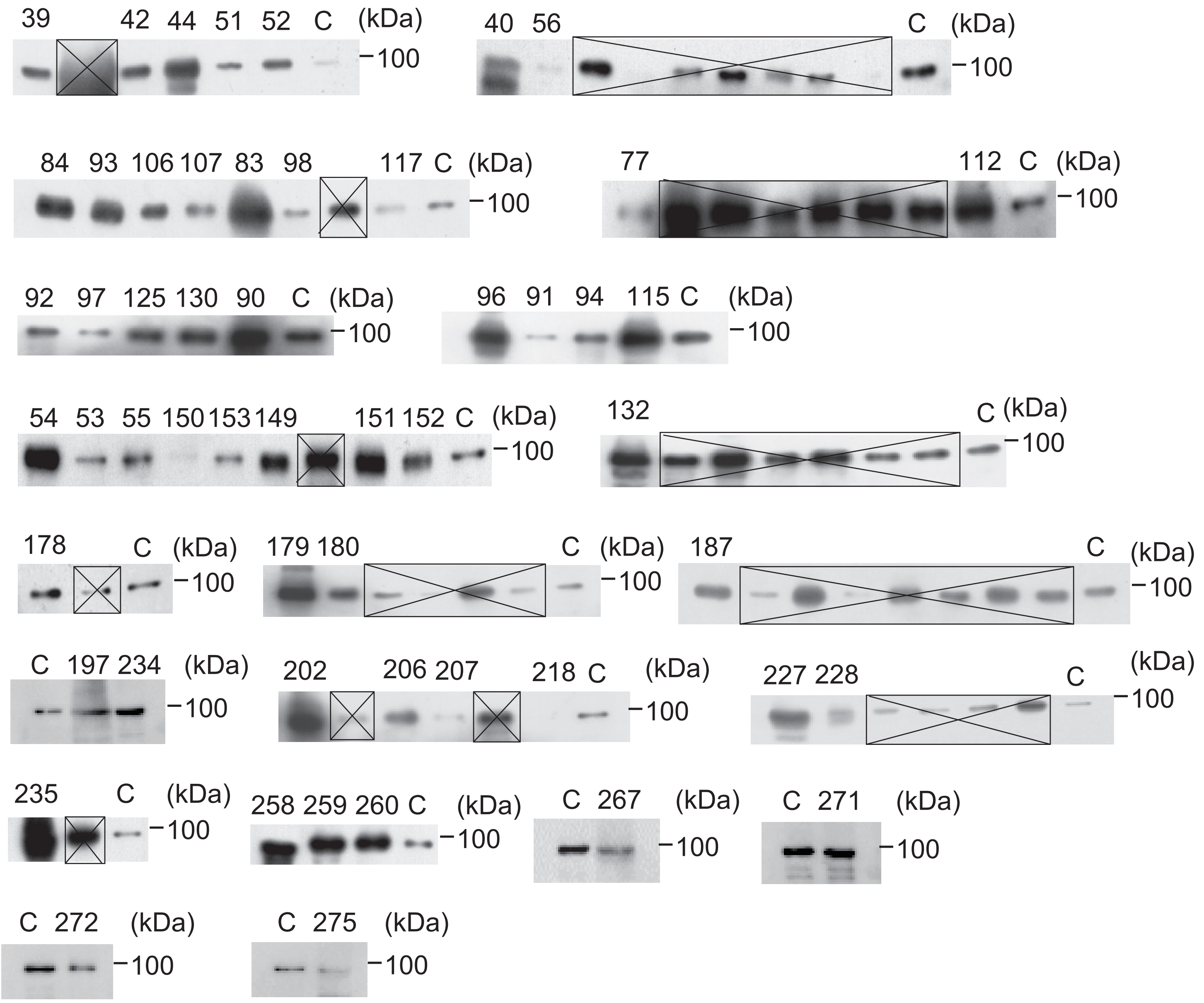

The cross mark is a sample (are samples) from other than the subject(s) of this research.

suppl Fig. 6 THP

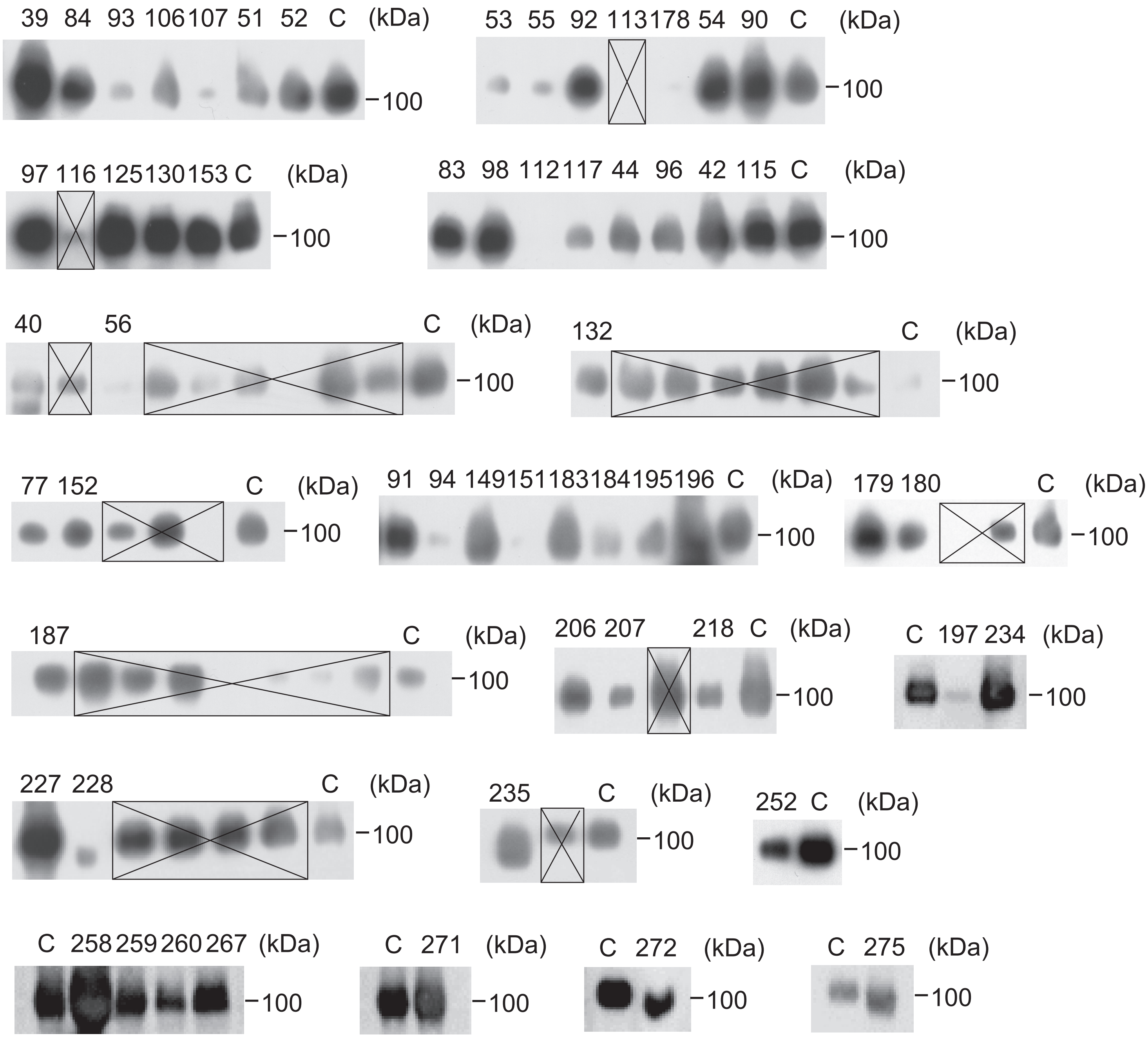

The cross mark is a sample (are samples) from other than the subject(s) of this research.
