# Supplementary material for: Reduced urinary release of AQP1‐ and AQP2‐bearing extracellular vesicles in patients with advanced chronic kidney disease
Source: Physiol Rep. 2021 Aug 26;9(17):e15005. doi: 10.14814/phy2.15005 (PMC8387789; doi:10.14814/phy2.15005)
Supplement: Supplementary file 3 — Table S1 [file PHY2-9-e15005-s003.pdf]

atient N	eGFR	CKD stage	administered drug								
245	3.8	5	alfacalcidol	telmisartan	sodium bicarbonate						
132	4.0	5	activated carbon	allopurinol	candesartan	sodium bicarbonate					
152	4.2	5									
220	4.4	5	tacrolimus	mycophenolate mofetil	benidipine	carvedilol	olmesartan	alfacalcidol	furosemide		
56	4.5	5	furosemide								
40	5.3	5									
77	5.7	5	amlodipine	olmesartan							
202	5.8	5	cilnidipine	candesartan	voglibose						
267	6.0	5	febuxostat	carvedilol	telmisartan	ethyl icosapentate	pravastatin	diclofenac			
187	6.4	5	tacrolimus	mycophenolate mofetil	alfacalcidol	losartan	enalapril	atorvastatin	allopurinol	benidipine	lafutidine
44	10.0	5	isosorbide mononi	metoprolol	candesartan	atorvastatin	amlodipine				
151	11.2	5	furosemide								
96	12.2	5	indapamide	olmesartan	amlodipine						
275	17.0	4	atorvastatin	febuxostat	amlodipine						
234	17.5	4	amlodipine	olmesartan							
197	19.7	4									
271	20.6	4	losartan	rosuvastatin	lansoprazole						
207	21.4	4	candesartan								
203	29.9	4	candesartan	cilnidipine							
179	31.5	3b									
259	33.3	3b	levothyroxine	ursodeoxycholic acid							
235	36.8	3b	amlodipine								
92	38.0	3b	enalapril	amlodipine							
243	39.2	3b	enalapril								
149	40.7	3b	candesartan	trichlormethiazide							
42	41.4	3b									
83	43.7	3b	diuretics								
125	44.2	3b	candesartan								
180	49.0	3a	candesartan	amlodipine							
130	49.0	3a									
52	49.5	3a									
94	50.2	3a	nifedipine	valsartan							
201	50.8	3a	levodopa	pergolide	theophylline	silodosin					
228	50.9	3a									
115	52.8	3a	amlodipine	olmesartan							
272	52.8	3a	candesartan	amlodipine							
227	54.7	3a									
90	55.2	3a	olopatadine	telmisartan							
112	55.6	3a									

106	56.2	3a	amlodipine	olmesartan	atorvastatin						
93	57.1	3a	olmesartan								
260	64.9	2	amlodipine	olmesartan							
229	67.8	2									
218	68.8	2									
153	70.6	2									
258	73.5	2	candesartan	furosemide							
51	80.6	2									
178	81.0	2									
91	81.1	2	amlodipine								
150	81.2	2									
54	82.2	2									
107	84.0	2	furosemide								
53	84.1	2									
117	89.8	2									
84	92.4	1									
97	101.9	1	sodium valproate	levetiracetam							
252	103.4	1									
39	105.8	1									
206	106.1	1									
208	109.4	1									
98	115.3	1									
55	126.0	1									
